# Mechanisms of Virtual Reality-Based Relaxation in Older Adults: A Scoping Review

**DOI:** 10.3390/jcm14176126

**Published:** 2025-08-29

**Authors:** Błażej Cieślik

**Affiliations:** Healthcare Innovation Technology Lab, IRCCS San Camillo Hospital, 30126 Venice, Italy; blazej.cieslik@hsancamillo.it

**Keywords:** virtual reality, elderly, relaxation, head-mounted display, physiological mechanisms, psychological mechanisms, gerontology, stress reduction, digital health

## Abstract

**Background/Objectives:** Mental health and emotional well-being are critical yet often overlooked in older adults. Immersive virtual reality (VR) may offer novel strategies for promoting relaxation in this population, but the diversity of intervention designs and underlying mechanisms remains insufficiently mapped. This scoping review aimed to systematically examine head-mounted display (HMD) VR interventions for relaxation in older adults, focusing on core scenarios, mechanisms, and conceptual paradigms. **Methods:** Following the JBI Manual for Evidence Synthesis and PRISMA-ScR guidelines, comprehensive searches of MEDLINE, Cochrane Library, Web of Science, and Scopus were conducted for empirical studies of HMD-based VR relaxation interventions in adults aged 60 and older. Data on study characteristics, hardware, intervention scenarios, and reported physiological and psychological mechanisms were extracted and categorized into conceptual paradigms. **Results:** Twenty-four studies were included, covering diverse clinical and community samples. Four main VR relaxation paradigms emerged: environmental relaxation, guided meditation, psychotherapy integration, and interactive gamified relaxation. Reported mechanisms included parasympathetic activation, multisensory immersion, attentional distraction, positive affect induction, mindfulness, cognitive engagement, and reminiscence activation. Considerable heterogeneity was found in intervention design and outcomes, with mechanisms often inferred rather than directly described. **Conclusions:** Similar relaxation mechanisms were engaged across paradigms, including hybrid interventions combining multiple approaches, highlighting the adaptability of VR to meet diverse needs. Identifying these four paradigms provides a foundation for future research and development of targeted VR-based relaxation for older adults. Further studies should clarify mechanisms, use standardized physiological outcome measures, and assess long-term benefits of VR relaxation interventions in geriatric populations.

## 1. Introduction

Mental health and emotional well-being are significant, yet often underrecognized, concerns in the aging population. Recent epidemiological data indicate that depression, anxiety, and stress affect 19.2%, 16.5%, and 13.9% of older adults, respectively [1]. Such mental health outcomes may be influenced by multiple factors, including chronic illness, declining functional capacity, reduced social networks, and major life transitions such as retirement or loss of independence [2,3]. Social isolation and loneliness, in particular, are associated with greater emotional distress and with the onset and persistence of depressive and anxiety symptoms in later life [4,5]. Collectively, these findings highlight the need for targeted strategies to address the psychosocial risks faced by older adults and promote mental health within this growing demographic.

In response to these challenges, recent systematic reviews underscore the importance of psychological resources such as emotional intelligence, adaptive coping, and social support in enhancing well-being and quality of life among older adults [6]. Beyond empirical effects, several established theories explain why relaxation works in this population. The relaxation response links paced breathing, progressive muscle relaxation, and mindfulness to parasympathetic downregulation and reduced stress arousal [7,8]. Models of attentional control and interoception account for shifts away from stressors toward bodily cues, while guided imagery engages associative affective pathways [9,10]. For nature-based methods, contemporary evidence shows that exposure to natural environments supports mood recovery and shifts in autonomic balance, consistent with restoration frameworks [11,12,13].

Consistent with this theoretical groundwork, nonpharmacological relaxation techniques, including progressive muscle relaxation, mindfulness, yoga, music therapy, guided imagery, and deep breathing, have gained recognition for reducing stress and supporting emotional health in older adults. A systematic review of 15 controlled trials demonstrated that such interventions significantly reduce depressive and anxiety symptoms, with some effects sustained for up to 24 weeks post-intervention [14]. Moreover, regular relaxation practice is associated with lower physiological dysregulation, particularly reduced inflammation, with benefits comparable to those achieved through exercise [15]. Techniques such as slow breathing, heart rate variability (HRV) biofeedback, and nature-based interventions have also shown promise in improving autonomic regulation and overall well-being in older adults [16,17].

Extending these mechanisms into a technology-mediated format, immersive virtual reality (VR) reproduces and, in some cases, amplifies relaxation through precise multisensory cueing within standardized, controllable settings [18]. VR systems range from nonimmersive desktop setups to fully immersive head-mounted displays (HMDs), offering graded levels of sensory engagement and interactivity [19,20]. Systematic reviews report that HMD-based VR relaxation interventions are generally feasible, well-accepted, and effective in reducing stress and promoting relaxation, particularly in general adult populations [21]. Immersive VR presentations of natural scenes, compared to indoor controls, have been shown to induce greater physiological relaxation, evidenced by decreased electrodermal activity (EDA), increased high-frequency heart rate variability (HF-HRV), and lower LF/HF ratios, all indicative of enhanced parasympathetic activity and reduced sympathetic arousal [22]. Riches and Williams (2025) further suggested that VR relaxation may be successfully implemented in hospital settings as a practical and portable intervention for reducing stress and anxiety among patients, families, and staff, especially when access to traditional relaxation spaces is limited [23]. Nevertheless, the effects and feasibility of HMD-VR relaxation interventions in older adults remain underexplored.

In a recent development, Montesano and Seinfeld (2025) introduced a three-dimensional framework for classifying VR interventions in psychotherapy, encompassing therapeutic strategy, psychological focus, and user perspective, with applications across diverse mental health conditions [24]. This framework considers all forms of immersive VR technology, not exclusively HMDs. While their focus is psychotherapeutic, the present review adopts a broader perspective, examining the full spectrum of VR-based relaxation methods relevant to older adults. Accordingly, the aim of this scoping review is to systematically map and synthesize the literature on HMD-VR interventions for relaxation in older adults, specifically (1) to describe the core scenarios utilized, (2) to delineate the principal physiological and psychological mechanisms of relaxation, and (3) to identify potential conceptual paradigms underlying these interventions.

## 2. Methods

### 2.1. Study Design

This scoping review was conducted in accordance with the methodological guidance outlined in Chapter 10.3 of the JBI Manual for Evidence Synthesis and the best practices recommended by Peters et al. [25]. Reporting followed the PRISMA Extension for Scoping Reviews (PRISMA-ScR) [26]; the completed checklist is provided in Supplementary File S1. The review protocol was registered a priori on the Open Science Framework (OSF; https://osf.io/5f6ba/).

### 2.2. Eligibility Criteria

Eligibility criteria were developed using the Population, Concept, Context (PCC) framework. Studies were eligible for inclusion if they (i) enrolled adults aged 60 years or older in any setting (community, clinical, or long-term care); (ii) evaluated HMD–VR interventions where the primary intent was relaxation (e.g., stress reduction, anxiety relief, mood improvement) in older adults; and (iii) reported physiological or psychological correlates of relaxation or provided sufficiently detailed descriptions of VR software or protocols to allow identification of these mechanisms.

Studies were excluded if (i) participants were younger than 60 years or age-specific results for participants ≥60 years could not be isolated; (ii) the intervention did not use HMD, including desktop/monitor-based virtual worlds, CAVE systems, augmented or mixed reality, non-immersive 360° video on flat screens, or smartphone apps without an HMD; or (iii) relaxation was not the primary aim or outcome, such as trials primarily targeting anxiety/phobias via virtual reality exposure therapy, pain management, addiction treatment, or functional/motor rehabilitation. Studies were also excluded if they were not primary empirical intervention reports (e.g., reviews, meta-analyses, theoretical/concept papers, protocols without results, case reports/series, qualitative-only studies, cross-sectional surveys, or purely observational designs). Only primary empirical intervention studies (e.g., randomized and nonrandomized trials, quasi-experimental, crossover, or single-arm pre–post designs) were eligible for inclusion; review and theoretical papers were excluded from synthesis but could be used for citation chaining. Age eligibility, HMD use, and relaxation focus were verified from the text; unclear cases followed predefined rules, and reasons for exclusion were logged (Supplementary File S3).

### 2.3. Information Sources, Search Strategy, and Screening

A literature search was conducted in July 2025 across four electronic databases: MEDLINE (via PubMed), Cochrane Library, Web of Science, and Scopus. The search strategy was developed according to the PCC framework. For the population component, search terms reflected older adults (e.g., aged, seniors, geriatric populations). For the concept, terms related to immersive virtual reality and HMD technologies were used. The context was addressed by including terms associated with relaxation, stress reduction, mindfulness, and related psychological outcomes. Terms identifying relevant study designs (e.g., randomized and non-randomized clinical trials) were also incorporated. The search strategy for each database was iteratively refined through pilot testing. Database selection was guided by coverage of biomedical and interdisciplinary sources, and a vocabulary-plus-keyword approach (controlled vocabulary plus free-text synonyms) was used to maximize retrieval; full, database-specific strings are provided in Supplementary File S2, PRISMA-ScR compliance is documented in Supplementary File S1, and the screening flow with exclusion reasons is detailed in Supplementary File S3. Citation chaining of included studies was also performed to ensure comprehensive coverage.

All identified records were deduplicated using the Deduplicator tool [27], part of the Systematic Review Accelerator suite, and subsequently managed in the Rayyan platform [28]. All steps of the screening process, including title and abstract screening, retrieval of potentially eligible records, and full-text assessment, were performed by the same reviewer (B.C.). Only articles published in English were included.

### 2.4. Data Extraction

Data were extracted by a single reviewer (B.C.) and compiled into a standardized extraction table. The following information was charted from each included study: authors, year of publication, sample characteristics, VR paradigm, type and level of interaction (active or passive), hardware used (HMD type), software or application, core scenario (i.e., content and procedure of the VR intervention), and the relaxation mechanisms involved (classified as physiological and psychological). Mechanisms were coded with a two-axis scheme: the evidence was marked as “M” (measured) when a mechanism-specific physiological or psychological correlate was reported (e.g., HRV/respiration, EEG alpha/theta, presence/immersion, imagery vividness, autonomy, or pre-specified affective targets such as state positive affect) and “H” (hypothesized) when no such correlate was collected. The source was given as “A” if stated by the primary authors and “R” if inferred by this review.

## 3. Results

### 3.1. Search Results

**Figure 1:**
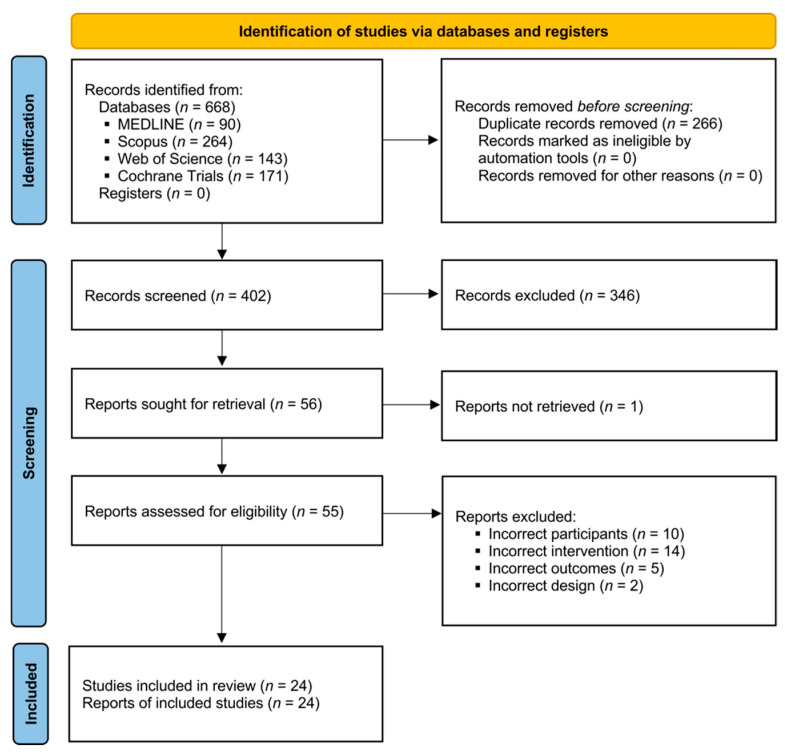
PRISMA flow diagram.

The initial search identified 90 articles from MEDLINE, 264 from Scopus, 143 from Web of Science, and 171 from the Cochrane Library. After deduplication, 402 unique records remained for title and abstract screening. Of these, 54 articles were selected for full-text review, resulting in the inclusion of 24 studies in the final synthesis (Figure 1). A list of excluded studies with reasons for exclusion is provided in Supplementary File S3.

### 3.2. Overview of Included Studies

A total of 24 studies were included, targeting various clinical and non-clinical older adult populations. These encompassed individuals with post-stroke conditions [29], Parkinson’s disease [30], cognitive impairment or dementia [31,32,33,34], cancer [35,36,37,38], insomnia [39], generalized anxiety disorder [40], and depressive symptoms [41], as well as intensive care unit patients [42] and community-dwelling or institutionalized older adults [43,44,45,46,47,48,49,50]. The characteristics of the included studies are summarized in Table 1.

Interaction types were either passive or active. Passive interactions, such as viewing immersive videos without user control, were used in 15 studies [31,32,33,34,36,37,38,39,40,42,43,44,48,49,50,51], while active interactions were applied in 9 studies and involved user manipulation or real-time engagement [29,30,40,41,45,46,47,50,52].

Most interventions employed standalone HMDs such as Oculus Quest/Quest 2 [47,49,52], Oculus Go [33,48] or HTC Vive [29,38,41]. Other configurations included Samsung Gear VR [31], smartphone-based HMDs [32,34,43,44], or custom solutions [35,36,45,50]. Software included both commercial platforms (YouTube VR) and bespoke applications designed for therapeutic purposes (VRTierOne, Joviality, VR-PHT-D1, Super Splendide).

### 3.3. Core Scenarios

Most studies were characterized by passive immersion in high-fidelity, naturalistic, or architecturally calming virtual environments. Participants typically viewed 360° nature scenes, gardens, forests, lakeshores, or culturally significant locations, frequently accompanied by ambient sounds such as birdsong, water, or soft music. The environmental content was often personalized, allowing participants to select or navigate to meaningful locations [35,38]. Some studies implemented facilitator-led group ‘tours’ of local landmarks and urban environments [43], while others focused on sequential passive viewing of immersive nature clips [31,32,34,36,37,42,44,49,51].

Psychotherapy-integrated scenarios embedded structured psychological interventions within the virtual environment. These approaches included reminiscence therapy using personalized 360° films, photo albums, and narrated cues designed to trigger autobiographical recall and positive affect [33,47,48]. Some studies featured virtual cognitive behavioral group therapy, mindfulness, or hypnotherapy exercises in a virtual group format [30,39]. In these interventions, the scenario served as a platform for therapist-guided or self-directed psychological tasks, including memory recall, role-play, cognitive restructuring, and graduated exposure.

Voice-guided meditative exercises formed the basis of the core scenario in several interventions. Participants engaged in passive, instructor-led meditation sessions, typically set within immersive naturalistic landscapes, with guidance on breathing, body scanning, compassion, or attention restoration [36,42,52]. In most cases, the meditation was delivered via pre-recorded audio, sometimes with minimal opportunities for scene or module selection.

Gamified relaxation scenarios aimed to actively engage participants in interactive VR tasks. These scenarios included activities such as virtual horticulture, in which users completed gardening-related tasks and received feedback and rewards [45], or VR aromatherapy training, where participants practiced oil mixing and anticipatory learning [50]. The interventions promoted cognitive engagement, a sense of mastery, and motivation through active participation.

Several studies adopted a hybrid scenario structure, combining elements of environmental relaxation, guided meditation, and psychotherapy. Examples include a platform that integrated interactive mandala coloring, soothing audio, and posthypnotic breathing guidance within a therapeutic virtual garden [29,41]. Other hybrid scenarios combined guided compassion meditation with active 360° nature exploration [46] or allowed the active selection of 360° relaxing videos with embedded diaphragmatic breathing coaching [35]. Scenarios were also developed that combined interactive open-world exploration with relaxation and guided imagery techniques [40] or merged narrated, music-accompanied views of familiar locations with reflection prompts for reminiscence and positive affect induction [48].

### 3.4. Relaxation Mechanisms

The reviewed studies employed a diverse set of mechanisms to facilitate relaxation, broadly categorized into physiological and psychological processes. The activation of these mechanisms was closely linked to the core scenario employed in each intervention.

Physiological mechanisms most commonly involved the induction of parasympathetic activity, often through paced or diaphragmatic breathing exercises. Such approaches were prominent in interventions incorporating guided meditation [36,42,52], voice-guided mindfulness sessions [39], and posthypnotic breathing cues [29,35,41,46]. Passive immersion in nature-rich or biophilic environments was also used to reduce sympathetic arousal and promote relaxation, with studies consistently reporting sensorial immersion as a key mechanism [31,32,34,37,38,43,44,49,51]. In several interventions, multisensory engagement, such as the integration of visual, auditory, and olfactory cues, was applied to deepen physiological relaxation [47,50].

Psychological mechanisms encompassed a range of cognitive and affective processes. Attentional distraction was a universally reported strategy, achieved through engaging virtual environments or tasks that absorbed participants’ focus and diverted attention from distressing symptoms or negative thoughts [31,32,34,36,37,38,42,43,44,49,51,52]. Many interventions also targeted positive affect induction, through either exposure to esthetically pleasing environments, personalized or meaningful content, or facilitated group discussions [32,43,47].

Specific psychological mechanisms were activated depending on scenario type. Mindfulness meditation and guided imagery were central in studies utilizing voice-guided meditation and relevant psychotherapy-integrated approaches [29,36,40,41,42,46,52]. Cognitive engagement and sense of mastery were emphasized in gamified and interactive scenarios, such as virtual horticulture and mandala coloring [29,40,41,45,50]. Reminiscence activation and autobiographical memory recall were core mechanisms in psychotherapy-integrated interventions focusing on reminiscence, leveraging familiar environments, personalized imagery, and narrative cues to elicit positive memories and emotional responses [33,47,48]. Additionally, some interventions facilitated cognitive restructuring, role-play, and graduated exposure as part of cognitive behavioral therapy modules [30,39].

### 3.5. Interconnections Between Paradigms and Mechanisms

**Figure 2:**
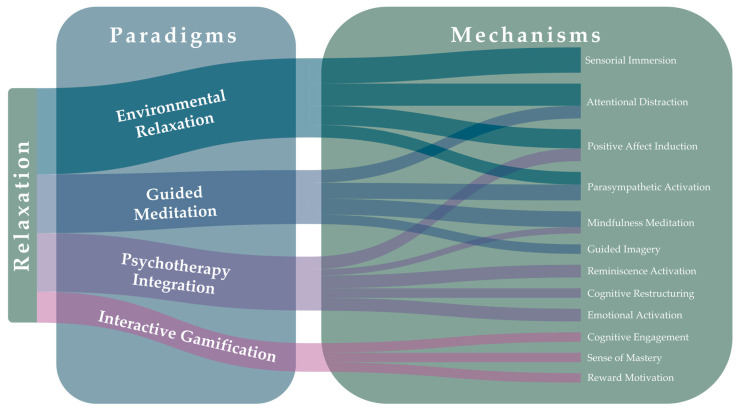
Alluvial mapping of VR relaxation mechanisms to paradigms.

To facilitate interpretation of relaxation mechanisms across heterogeneous interventions, the included studies were categorized into four conceptual paradigms. The classification was developed post hoc through inductive analysis and was based on shared features in content structure and therapeutic objectives. The paradigms are defined below and illustrated in Figure 2:

▪ Interventions rely on exposure to calming virtual environments and do not include a scripted meditation or a psychotherapy protocol. The primary mechanism is attention restoration (“soft fascination”) and affective soothing from scenic content, consistent with Attention Restoration Theory [53,54]. Typical examples are passive 360° nature [34] or cultural scenes used in acute and long-term care [37].
▪ Guided-Meditation Modules: The active ingredient is a scripted contemplative practice, such as breath focus, body scanning, or guided imagery, delivered by narration and without a manualized psychotherapy protocol. Mechanistically, these interventions target top–down attentional and interoceptive regulation and can promote parasympathetic calming (relaxation response; mindfulness models). Examples include voice-guided meditation presented within immersive nature scenes [36,42].
▪ Psychotherapy-Integrated: VR is used to deliver a named, manualizable psychotherapeutic approach (e.g., CBT with exposure/restructuring, hypnotherapy, reminiscence, compassion training). Change is driven by protocol-specific processes such as exposure and extinction, cognitive reappraisal, autobiographical reactivation, or hypnotic suggestion. Examples include VR-CBGT for Parkinson’s disease [30] and personalized 360° reminiscence sessions [47].
▪ Interactive Gamified Relaxation: Interventions center on goal-directed interaction built on game mechanics, rules and challenges, levels, points/rewards, and performance feedback, aimed at eliciting relaxation through mastery, competence, and flow (Self-Determination Theory and flow theory) [55,56]. Illustrative examples are virtual horticulture with rewards [45] and interactive mandala coloring [41].

## 4. Discussion

This review systematically mapped the landscape of HMD-VR interventions for relaxation in older adults. The findings reveal considerable diversity in intervention design, including four main conceptual paradigms: immersive environmental exposure, guided meditation, psychotherapy integration, and gamified activities. Across these approaches, interventions consistently engaged both physiological and psychological mechanisms, with sensorial immersion and attentional distraction emerging as fundamental components. More structured or interactive scenarios additionally targeted mindfulness, reminiscence, or cognitive engagement.

All included interventions used HMDs, providing a common baseline of system-level immersion. Immersion and presence are distinct; presence is shaped not only by HMDs but also by field of view, stereoscopy, tracking, sensory richness, interactivity, latency, and user control [57,58]. In 2015, Cummings and Bailenson showed that greater immersion, achieved through wider field of view, stereoscopic display, and robust tracking, tends to increase presence, which can amplify cognitive and affective effects [59]. A recent study further demonstrates that deeply immersive environments can enhance memory, engagement, and task performance, offering methodological cues (e.g., richer multisensory cues and meaningful interactivity) that may translate to stronger relaxation effects in older adults [60]. At the same time, VR design must balance presence with cognitive load and usability to avoid counterproductive effects [60]. Taken together, these findings suggest that, beyond the mere use of HMDs, immersive features such as sensory richness and meaningful interactivity likely shape relaxation responses via their impact on presence and engagement.

### 4.1. Paradigms and Mechanisms

The identification of four conceptual paradigms clarified the shared and distinct mechanisms underlying VR-based relaxation. This categorization demonstrates that HMD-VR interventions for older adults are flexible and multi-component, supporting a wide range of psychological and physiological outcomes adapted to user needs and context.

Among these paradigms, immersive environmental relaxation was most frequently implemented. Robust evidence from both VR and non-VR research consistently demonstrates that exposure to natural environments facilitates mood improvement, anxiety reduction, and favorable modulation of physiological parameters such as cardiac and autonomic activity [61,62]. Passive visual exposure to nature reliably induces relaxation, and real-life greenery enhances prefrontal cortex activation [63]. By providing immersive access to tranquil natural settings that might otherwise be inaccessible for older adults, VR-based interventions have been shown to evoke tranquility and positive affect and to reduce anxiety [64]. Neurophysiological studies further indicate modulation of electroencephalography (EEG) features associated with relaxation [65], suggesting potential for future neuroadaptive interventions. Notably, evidence from real-world immersive environments also suggests that physiological responses are not always unidirectional; increases in heart rate and decreases in heart rate variability may occur alongside improved mood and stress perception, possibly reflecting heightened attentional or emotional engagement [11].

Guided meditation modules constituted another major paradigm in the reviewed interventions. These approaches leverage immersive VR environments, spatialized audio, and embodied cues to facilitate deep engagement in meditative practice. Evidence indicates that VR-guided meditation acutely modulates autonomic and cortical activity, resulting in reduced physiological arousal, such as lower heart rate and cortisol levels, without causing discomfort [66]. Innovative uses of VR allow for manipulation of the sense of self, with different perspectives during meditation inducing sensations of detachment, reduced self-identification with the body, and measurable neural changes in brain areas linked to bodily self-awareness [67]. Both VR- and imagery-based guided meditation have been shown to significantly decrease physiological arousal and negative affect, though VR tends to enhance concentration and positive emotions, particularly among novice meditators [68].

Psychotherapy-integrated VR relaxation interventions represent a paradigm in which established psychotherapeutic methods are delivered within immersive virtual environments to address both emotional and cognitive aspects of well-being. The reviewed studies included a broad range of psychotherapies delivered via VR, such as mindfulness-based approaches, hypnotherapy, cognitive behavioral group therapy, compassion-focused interventions, guided imagery, and reminiscence therapy. For example, one approach used a virtual therapeutic garden with soothing music and posthypnotic breathing guidance, based on Eriksonian psychotherapy principles [29,41]. Although the theoretical background for using hypnosis in virtual reality was proposed almost 16 years ago, systematic research on VR-based hypnotherapy has only emerged recently [69]. Recent analyses highlight a growing interest in technology-enhanced hypnotherapy, including applications of Eriksonian techniques for a range of psychological and somatic conditions, and a recent meta-analysis demonstrates that virtual reality-enhanced hypnosis is particularly effective in reducing anxiety, pain, and physiological stress responses [70] and should be prioritized over passive, audio-only formats whenever possible [71].

Other studies reported the use of structured group-based cognitive behavioral and mindfulness exercises, including exposure therapy, delivered through custom VR platforms [30]. A recent scoping review found that cognitive behavioral therapy, including internet-based and VR-assisted formats, was commonly used to address psychological distress during the COVID-19 pandemic, with VR identified as a promising alternative when in-person therapy was unavailable [72]. Additionally, guided compassion meditation and mindfulness-based compassion practices were delivered in VR, supporting both parasympathetic activation and emotional regulation [39,46,52]. Unlike stand-alone guided meditation modules, these psychotherapy-integrated interventions use mindfulness as part of a broader therapeutic framework to address specific psychological processes and enhance long-term well-being. Qualitative findings indicate that VR-based practices are perceived as more accessible and engaging and less effortful than traditional mindfulness, with potential benefits for both acute relief and preventive self-regulation [73]. However, quantitative results are still forthcoming, as the meta-analysis protocol has only recently been registered [74].

Reminiscence therapy was also represented among the psychotherapy-integrated interventions, making use of personalized 360° VR environments and interactive photo albums to support autobiographical memory recall and emotional engagement [33,47,48]. Recent studies suggest that such VR-based reminiscence approaches may help sustain or modestly improve cognitive function during active use, with consistent benefits observed in emotional well-being and user engagement, especially when the scenarios are immersive and tailored to individual experiences [75,76].

The final conceptual paradigm identified was interactive gamified relaxation, which incorporates game-like or creative tasks within immersive VR environments to enhance engagement and motivation. Examples include virtual horticulture with reward systems and interactive mandala coloring in a virtual garden [29,41,45]. These are classic gamification mechanisms used to promote active participation and positive affect [77]. Recent frameworks, such as the one proposed by Kim and Choi (2025), highlight the potential of integrating gamification and multimodal arts, such as music, visual art, and interactive storytelling, into VR-based digital therapeutics to further increase user engagement, emotional immersion, and skill development [78]. Such innovations not only support active participation and positive affect but also enable real-time data collection for the assessment of both clinical and user experience outcomes.

### 4.2. Bigger Picture

Positioning VR relaxation within digital therapeutics highlights both opportunities and evidentiary gaps. Software-based interventions should demonstrate clinical validity, usability, and real-world adherence, not just engaging content. From cognitive psychology, the core processes overlap with conventional relaxation: attentional control and disengagement from stressors; interoceptive focus (e.g., breathing); guided imagery; and exposure to restorative cues. VR may amplify these through presence, multisensory richness, and interactive agency [79]. Neurophysiologically, markers such as HRV, respiration/EDA, EEG alpha and theta activity, and salivary cortisol are relevant in both VR and non-VR formats [80,81,82]. VR also introduces factors that can moderate benefits in older adults, including cybersickness and cognitive workload [83]. Compared with standard relaxation and mindfulness programs, VR may improve access and engagement by transporting users to otherwise inaccessible environments [84]. However, evidence for superiority remains preliminary and context-dependent.

Beyond clinical and technical parameters, VR relaxation for older adults can be viewed as a sociotechnical intervention shaped by context. Adoption, engagement, and outcomes are influenced by digital health literacy and prior exposure to digital media, so assessing literacy with age-appropriate instruments is advisable [85]. Qualitative work in aged-care settings reports both opportunities and concerns, from curiosity and perceived therapeutic potential to questions about authenticity, cognitive demands, safety, and the need for supervision [86]. Ethical analyses further emphasize autonomy, informed consent, privacy, and the avoidance of manipulative design, which is especially salient for vulnerable users. Social VR can support connection and engagement when usability and presence are prioritized, while practical design needs such as training time, avatar realism, and controller ergonomics affect acceptability [87]. Accordingly, studies should report digital literacy or technology acceptance and access barriers and incorporate culturally responsive content, age-friendly interfaces, and caregiver involvement to support equitable implementation [88].

### 4.3. Strengths and Limitations

This review has several methodological strengths. It adhered to JBI guidance and PRISMA-ScR reporting, with a preregistered protocol. A multi-database, vocabulary-plus-keyword search with citation chaining was implemented; full search strings, checklists, and reasons for exclusion are provided in Supplementary Files. Data were charted using standardized fields, conceptual paradigms were defined with clear operational rules and precedence, and mechanisms were annotated with a two-axis scheme (measured vs. hypothesized; author-stated vs. review-inferred) to enhance transparency.

These strengths are tempered by important limitations. First, the literature search and data extraction were performed by a single reviewer, which may increase the risk of selection and extraction bias despite the use of standardized forms. Second, most included studies were small and methodologically heterogeneous and reported varied outcome measures, limiting generalizability and complicating cross-study comparisons. Third, qualitative-only and observational designs were excluded, and given the subjective and experiential nature of immersive VR, this limits insights into user perceptions, emotions, and meaning-making. Finally, many studies did not provide clear descriptions or theoretical rationales for the relaxation mechanisms underlying their interventions. Consequently, the mechanisms often had to be inferred based on available details about procedures, software content, or the stated aims of the intervention rather than being systematically described by the study authors. This reliance on interpretation may have affected the accuracy and consistency of how mechanisms were classified in this review.

### 4.4. Implications and Future Study Directions

By identifying core scenarios, mechanisms, and conceptual paradigms, this review offers a preliminary, hypothesis-generating framework to inform the design of VR relaxation interventions. Given the heterogeneity of study designs, samples, outcomes, and follow-up, the applicability of these insights to practice is limited, and any recommendations should be viewed as provisional rather than prescriptive. In clinical settings, VR-based relaxation may be considered an adjunct to traditional approaches, particularly where access to trained facilitators is limited or patient engagement is low, pending confirmatory evidence. The conceptual mapping presented here may inform the development of user-centered VR solutions and guide implementation in clinical and community contexts. Finally, this study underscores the importance of clearly documenting intervention mechanisms and rationales in future research to advance the development and evaluation of VR-based relaxation strategies.

From a VR system development perspective, several directions remain promising yet exploratory: (i) acoustic neural modulation, in which background music is engineered with specific amplitude or frequency modulations to support relaxation and attentional control [89,90,91]; (ii) closed-loop HRV biofeedback embedded in immersive nature settings, which in early studies has improved autonomic indices and psychological outcomes such as relaxation self-efficacy, attentional focus, and engagement [92]; and (iii) VR-based biofeedback using EEG, galvanic skin response, respiratory feedback, or mindfulness prompts, with preliminary reductions reported in biochemical stress markers and perceived strain or fatigue [93].

From a clinical study perspective, future research should include objective physiological indices (e.g., HRV, salivary cortisol) alongside multimodal neurophysiology/neuroimaging (EEG/fMRI) and validated psychological measures to characterize relaxation effects in older adults. Mechanistic hypotheses should be evaluated using preregistered mediation analyses. Comparing changes across candidate paradigms could help clarify possible pathways, but definitive comparative effectiveness remains uncertain. In parallel, network meta-analysis could be applied to systematically compare the effectiveness of various approaches and paradigms, recognizing that simpler interventions like environmental relaxation may be less resource-intensive than more complex modalities such as psychotherapy-integrated VR. Because these findings are context-dependent and derived from heterogeneous, often small trials, they should be considered hypothesis-generating. For the next phase, adequately powered confirmatory studies should standardize outcomes and usability assessments, and include comparisons with simpler exposure-based VR and active non-VR comparators. They should also prioritize adaptive personalization tailored to cognitive status, sensory and mobility impairments, and user preferences, with predefined 3-, 6-, and 12-month follow-ups. Economic evaluation should be embedded, reporting trial- and model-based cost-effectiveness.

## 5. Conclusions

In conclusion, this review identified that head-mounted display virtual reality interventions for relaxation in older adults employed a range of core scenarios. Each type of intervention engaged both physiological and psychological mechanisms, most notably sensory immersion, parasympathetic activation, attentional redirection, and positive affect induction. While sensory immersion and attentional redirection represented foundational elements across all paradigms, more structured interventions further leveraged mechanisms such as mindfulness, cognitive engagement, reminiscence, or mastery.

The analysis of conceptual paradigms suggested that similar relaxation mechanisms were often engaged across different scenario types, which may indicate that VR interventions can be adapted to address diverse user needs and therapeutic goals. By identifying four main paradigms, this review offers a working conceptual framework to guide future research and the development of tailored VR-based interventions for older adults.

## Figures and Tables

**Table 1:** Key characteristics of included studies on HMD-VR relaxation interventions.

Study	Population	Paradigm	Hardware	Software/App	Core Scenario	Mechanisms
Malbos et al. (2025) [40]	58 patients with GAD	Hybrid: Environmental Relaxation; Psychotherapy-Integrated (Guided Imagery)	Sensics zSight HMD	CryEngine Sandbox–based virtual environments	Interactive open-world VR with customizable environments (islands, forests) and conditions (weather, music)	Physiological: Parasympathetic activation [M/R]; sensorial immersion [M/A] Psychological: Guided imagery [H/A]; attentional distraction [H/A]; autonomy support through interactive exploration and customizable scenarios [H/A]
Tayyebi et al. (2025) [30]	90 Parkinson’s patients	Psychotherapy-Integrated (Cognitive Behavioral Group Therapy, Mindfulness)	Unspecified HMD	Custom VR-CBGT platform	Active participation in group VR mindfulness and cognitive-behavioral exercises, including exposure therapy	Physiological: Parasympathetic activation via paced breathing [M/A] Psychological: Mindfulness [H/A]; cognitive restructuring [H/A]; role-play; graduated exposure [H/A]
Sadowski et al. (2025) [46]	24 older adults	Hybrid: Environmental Relaxation; Psychotherapy-Integrated (Compassion)	Meta-Quest 2 HMD	Super Splendide EEMCF-VR app	Guided compassion mindfulness meditation followed by active 360° nature exploration	Physiological: Parasympathetic activation [H/R] Psychological: Mindfulness meditation [M/A]; compassion practice [H/R]
Wu et al. (2025) [36]	118 breast cancer patients	Guided Meditation Modules	Unspecified HMD	Custom mind–body interactive VR system	Passive voice-guided meditation in immersive natural landscapes	Physiological: Parasympathetic activation via paced breathing [H/A]; sensorial immersion [H/A] Psychological: Guided imagery [H/R]; attentional distraction [H/A]
Şansal et al. (2024) [44]	60 older adults	Environmental Relaxation	Smartphone-in-goggles VR system	360° videos (therapy forest, urban scenes)	Passive seated viewing of static 360° therapeutic nature scenes featuring vegetation, water, and animals	Physiological: Sensorial immersion reducing sympathetic arousal [H/R] Psychological: Attentional distraction [H/R]; positive affect induction [M/A]
Abd El Fatah et al. (2024) [47]	60 older adults in assisted living facilities	Psychotherapy-Integrated (Reminiscence Therapy)	Oculus Quest HMD	VR Wander (Google Street Tour) + bespoke VR photo-albums and videos	Personalized active reminiscence VR sessions exploring hometowns and interacting with life-history images and narrated cues	Physiological: Multisensory engagement via interactive audiovisual stimuli [M/A] Psychological: Reminiscence activation [H/A]; autobiographical memory recall [H/A]; positive affect induction through personalized familiar content [H/A]
Wan et al. (2024) [39]	63 chronic insomnia patients	Psychotherapy-Integrated (Mindfulness, Hypnotherapy)	All-in-one VR headset (VR-PHT-D1 system)	VR-PHT-D1 Mental Health Training System	Passive viewing of themed relaxation–mindfulness–hypnotherapy scenarios	Physiological: Parasympathetic activation via paced breathing; sensorial immersion [M/R] Psychological: Mindfulness meditation [H/A]; hypnotic suggestion [H/A]; attentional distraction [H/A]
Woo et al. (2024) [35]	128 terminal cancer patients	Hybrid: Environmental Relaxation; Psychotherapy-Integrated (Attention Restoration); Guided Meditation Modules	Meta Quest 2 HMD	YouTube VR 360° relaxation videos	Active selection of 360° relaxing videos with diaphragmatic breathing coaching	Physiological: Parasympathetic activation via diaphragmatic breathing; sensorial immersion [H/A] Psychological: Attentional distraction; attention restoration [H/A]; positive affect induction [H/A]; flow induction; [H/A] autonomy support through personalized content selection [H/A]
Xiaoxue & Huang (2024) [51]	23 older adults	Environmental Relaxation	Unspecified HMD)	SketchUp built environments rendered in Enscape v3.4	Passive immersion in biophilic VR scenes (gardens, nature-built spaces)	Physiological: Parasympathetic activation through biophilic visual stimuli [M/A]; sensorial immersion [H/A] Psychological: Attentional distraction [H/A]; positive affect induction [M/A]
Burrows et al. (2023) [52]	10 hemodialysis patients	Hybrid: Guided-Meditation Modules; Psychotherapy-Integrated (Mindfulness)	Oculus Quest 2 HMD	Guided Meditation VR app	Passive mindfulness meditation experience combined with immersive nature environments	Physiological: Parasympathetic activation through mindfulness meditation [H/A] Psychological: Mindfulness meditation (body scanning, breath focus) [M/A]; attentional distraction [H/A]; emotional activation via immersive nature exposure [H/A]
Cieślik et al. (2023) [41]	60 elderly women with depressive symptoms	Hybrid: Environmental Relaxation; Psychotherapy-Integrated (Hypnotherapy); Interactive Gamified Relaxation	HTC VIVE	VRTierOne	Active mandala coloring in a virtual therapeutic garden with soothing music and posthypnotic breathing guidance	Physiological: Parasympathetic activation via paced breathing [H/R] Psychological: Guided imagery [H/R]; cognitive engagement through interactive mandala coloring; relaxation induction via posthypnotic suggestion [H/R]
Moon et al. (2023) [37]	58 palliative care patients	Environmental Relaxation	PICO Neo 3 Pro 5.7K VR headset	Tablet-controlled motus VR system with bespoke 360° videos	Passive, patient-selected immersive 360° nature and cultural scenes (e.g., boat trips, kayaking)	Physiological: Sensorial immersion reducing sympathetic arousal [H/A] Psychological: Attentional distraction [H/A]; positive affect induction [H/A]; autonomy support through personalized content selection [H/A]
Appel et al. (2022) [33]	33 veterans with dementia	Hybrid: Environmental Relaxation; Psychotherapy-Integrated (Reminiscence)	Oculus Go HMD	360° VR film library + bespoke veteran-relevant 360° videos	Passive viewing of personalized 360° films triggering reminiscence	Physiological: Sensorial immersion [H/R] Psychological: Attentional distraction [H/R]; positive affect induction [M/A]; reminiscence triggering [M/A]
Chaze et al. (2022) [48]	32 long-term-care residents	Hybrid: Environmental Relaxation; Guided Meditation Modules; Psychotherapy-Integrated (Reminiscence)	Oculus Go HMD	Narrated 360° Canadian landmarks	Passive viewing of narrated, music-accompanied familiar locations with brief reflection prompts	Physiological: Sensorial immersion reducing sympathetic arousal [H/R] Psychological: Guided imagery [H/R]; reminiscence activation [M/A]; attentional distraction [M/A]; positive affect induction [M/A]
Gruber et al. (2022) [49]	17 nursing-facility residents	Environmental Relaxation	Oculus Quest	360° video library (diginetmedia, 10 videos)	Passive viewing of categorized 360° nature and activity-based videos as care supplement	Physiological: Sensorial immersion reducing sympathetic arousal [H/R] Psychological: Attentional distraction [H/R]; positive affect induction [M/A]
Fan et al. (2022) [45]	62 older adults	Interactive Gamified Relaxation	Unspecified 3D-VR helmet	Custom 3D-VR horticultural simulation	Active virtual horticulture tasks with rewards, paired with physical gardening tasks	Physiological: Active engagement [H/A]; sensorial immersion [H/A] Psychological: Sense of mastery [M/A]; reward motivation [M/A]; positive affect induction [H/A]
Kiper et al. (2022) [29]	60 post-stroke survivors	Hybrid: Environmental Relaxation; Psychotherapy-Integrated (Hypnotherapy); Interactive Gamified Relaxation	HTC Vive Pro	VRTierOne	Active mandala coloring in a virtual therapeutic garden with soothing music and posthypnotic breathing guidance	Physiological: Parasympathetic activation via paced breathing [H/A] Psychological: Guided imagery [H/A]; cognitive engagement through interactive mandala coloring [H/A]; relaxation induction via posthypnotic suggestion [H/A]
Appel et al. (2021) [31]	10 dementia inpatients	Environmental Relaxation	Samsung Gear VR HMD	Custom 360° nature video app	Passive viewing of immersive 360° nature clips (lakeshore, forest, icebergs)	Physiological: Sensorial immersion reducing sympathetic arousal [H/R] Psychological: Attentional distraction [M/A]; positive affect induction [M/A]
Brimelow et al. (2021) [32]	25 residential aged-care residents	Environmental Relaxation	Samsung Galaxy S7 + Gear VR wireless HMD	Off-the-shelf 360° video apps	Active selection and viewing of 360° nature, landmark, and historical videos with facilitator-led debriefing	Physiological: Sensorial immersion reducing sympathetic arousal [H/A] Psychological: Attentional distraction [H/A]; positive affect induction [M/A]; social interaction through facilitated discussions [H/A]
Appel et al. (2020) [34]	66 older adults	Environmental Relaxation	Samsung S7 smartphone + Gear VR HMD	Custom 360° nature video collection	Passive viewing of sequential 360° immersive nature clips	Physiological: Sensorial immersion reducing sympathetic arousal [H/A] Psychological: Attentional distraction [H/A]; positive affect induction [H/A]
Chan et al. (2020) [43]	236 older adults	Environmental Relaxation	Smartphone-based HMD (custom headset)	Tailored mobile VR app (360° Hong Kong scenes)	Active VR “tours” of Hong Kong landmarks, with facilitator-led discussions between clips	Physiological: Sensorial immersion reducing sympathetic arousal [H/A] Psychological: Attentional distraction [H/A]; positive affect induction [M/A]; social engagement via group discussion [H/A]
Cheng et al. (2020) [50]	60 institutionalized older adults	Multisensory Therapy-Integrated (Aromatherapy Training)	Unspecified HMD	Custom 3D VR aromatherapy app	Active VR aromatherapy training and oil-mixing practice	Physiological: Multisensory engagement (visual and olfactory stimulation) [H/A] Psychological: Emotional activation [M/A]; anticipatory learning and cognitive engagement through aromatherapy training tasks [H/A]
Lee & Kang (2020) [42]	48 intensive care unit patients	Guided Meditation Modules	Unspecified HMD	Custom VR meditation program	Passive voice-guided meditation with immersive nature scenes and paced breathing prompts	Physiological: Parasympathetic activation via paced breathing [H/R]; sensorial immersion [H/A] Psychological: Mindfulness meditation [H/A]; attentional distraction [H/A]
Niki et al. (2019) [38]	20 terminal cancer patients	Environmental Relaxation	HTC VIVE headset	Google Earth VR	Passive self-navigation of personalized 360° VR travel to meaningful locations	Physiological: Sensorial immersion reducing sympathetic arousal [H/A] Psychological: Attentional distraction [H/A]; positive affect induction [H/A]; autobiographical memory activation through personally meaningful locations [H/A]

HMD, head-mounted display; VR, virtual reality; GAD, generalized anxiety disorder. Mechanism tags use [Evidence/Source]: M, measured (mechanism-specific physiological/psychological correlate reported); H, hypothesized (no mechanism-specific correlate); A, stated by the primary authors; R, inferred by this review.

## Data Availability

All data generated and analyzed during this review are included in this manuscript and its Supplementary Materials.

## Supplementary Materials

The following supporting information can be downloaded at https://www.mdpi.com/article/10.3390/jcm14176126/s1, Supplementary File S1: PRISMA checklist; Supplementary File S2: Search strategy for databases; Supplementary File S3: List of excluded studies with reasons.

## References

[1] Jalali A., Ziapour A., Karimi Z., Rezaei M., Emami B., Kalhori R.P., Khosravi F., Sameni J.S., Kazeminia M. (2024). Global Prevalence of Depression, Anxiety, and Stress in the Elderly Population: A Systematic Review and Meta-Analysis. BMC Geriatr..

[2] Fiske A., Wetherell J.L., Gatz M. (2009). Depression in Older Adults. Annu. Rev. Clin. Psychol..

[3] Hermsen L.A.H., Smalbrugge M., van der Wouden J.C., Leone S.S., Dekker J., van der Horst H.E. (2014). Trajectories of Physical Functioning and Their Prognostic Indicators: A Prospective Cohort Study in Older Adults with Joint Pain and Comorbidity. Maturitas.

[4] Hajek A., Sutin A.R., Posi G., Stephan Y., Peltzer K., Terracciano A., Luchetti M., König H.-H. (2025). Chronic Loneliness and Chronic Social Isolation among Older Adults. A Systematic Review, Meta-Analysis and Meta-Regression. Aging Ment. Health.

[5] Courtin E., Knapp M. (2017). Social Isolation, Loneliness and Health in Old Age: A Scoping Review. Health Soc. Care Community.

[6] Frías-Luque M.D., Toledano-González A. (2022). Determinants of Quality of Life and Well-Being in Cognitively Unimpaired Older Adults: A Systematic Review. PeerJ.

[7] Lehrer P.M., Gevirtz R. (2014). Heart Rate Variability Biofeedback: How and Why Does It Work?. Front. Psychol..

[8] Groß D., Kohlmann C.-W. (2021). Increasing Heart Rate Variability through Progressive Muscle Relaxation and Breathing: A 77-Day Pilot Study with Daily Ambulatory Assessment. Int. J. Environ. Res. Public Health.

[9] Holmes E.A., Mathews A. (2010). Mental Imagery in Emotion and Emotional Disorders. Clin. Psychol. Rev..

[10] Eysenck M.W., Derakshan N., Santos R., Calvo M.G. (2007). Anxiety and Cognitive Performance: Attentional Control Theory. Emotion.

[11] Scott E.E., LoTemplio S.B., McDonnell A.S., McNay G.D., Greenberg K., McKinney T., Uchino B.N., Strayer D.L. (2021). The Autonomic Nervous System in Its Natural Environment: Immersion in Nature Is Associated with Changes in Heart Rate and Heart Rate Variability. Psychophysiology.

[12] Farrow M.R., Washburn K. (2019). A Review of Field Experiments on the Effect of Forest Bathing on Anxiety and Heart Rate Variability. Glob. Adv. Health Med..

[13] Fan L., Baharum M.R. (2024). The Effects of Digital Nature and Actual Nature on Stress Reduction: A Meta-Analysis and Systematic Review. Internet Interv..

[14] Klainin-Yobas P., Oo W.N., Suzanne Yew P.Y., Lau Y. (2015). Effects of Relaxation Interventions on Depression and Anxiety among Older Adults: A Systematic Review. Aging Ment. Health.

[15] Glei D.A., Goldman N., Lin Y.-H., Weinstein M. (2012). Relaxation Practice and Physiologic Regulation in a National Sample of Older Taiwanese. J. Altern. Complement. Med..

[16] Giorgi F., Tedeschi R. (2025). Breathe Better, Live Better: The Science of Slow Breathing and Heart Rate Variability. Acta Neurol. Belg..

[17] Tong K., Thompson C.W., Carin-Levy G., Liddle J., Morton S., Mead G.E. (2025). Nature-Based Interventions for Older Adults: A Systematic Review of Intervention Types and Methods, Health Effects and Pathways. Age Ageing.

[18] Helou S., Khalil N., Daou M., El Helou E. (2023). Virtual Reality for Healthcare: A Scoping Review of Commercially Available Applications for Head-Mounted Displays. Digit. Health.

[19] Slater M., Sanchez-Vives M.V. (2016). Enhancing Our Lives with Immersive Virtual Reality. Front. Robot. AI.

[20] Wiederhold B.K., Wiederhold M.D. (2005). Virtual Reality Therapy for Anxiety Disorders: Advances in Evaluation and Treatment.

[21] Riches S., Azevedo L., Bird L., Pisani S., Valmaggia L. (2021). Virtual Reality Relaxation for the General Population: A Systematic Review. Soc. Psychiatry Psychiatr. Epidemiol..

[22] Anderson A.P., Mayer M.D., Fellows A.M., Cowan D.R., Hegel M.T., Buckey J.C. (2017). Relaxation with Immersive Natural Scenes Presented Using Virtual Reality. Aerosp. Med. Hum. Perform..

[23] Riches S., Williams G. (2025). Virtual Reality Relaxation for Hospitals: A Novel Stress-Reduction Intervention for Patients, Families, and Staff. Future Healthc. J..

[24] Montesano A., Seinfeld S. (2025). Virtual Reality in Psychotherapy: A Three-Dimensional Framework to Navigate Immersive Clinical Applications. J. Clin. Psychol..

[25] Peters M.D.J., Marnie C., Tricco A.C., Pollock D., Munn Z., Alexander L., McInerney P., Godfrey C.M., Khalil H. (2020). Updated Methodological Guidance for the Conduct of Scoping Reviews. JBI Evid. Synth..

[26] Tricco A.C., Lillie E., Zarin W., O’Brien K.K., Colquhoun H., Levac D., Moher D., Peters M.D.J., Horsley T., Weeks L. (2018). PRISMA Extension for Scoping Reviews (PRISMA-ScR): Checklist and Explanation. Ann. Intern. Med..

[27] Forbes C., Greenwood H., Carter M., Clark J. (2024). Automation of Duplicate Record Detection for Systematic Reviews: Deduplicator. Syst. Rev..

[28] Ouzzani M., Hammady H., Fedorowicz Z., Elmagarmid A. (2016). Rayyan-a Web and Mobile App for Systematic Reviews. Syst. Rev..

[29] Kiper P., Przysiężna E., Cieślik B., Broniec-Siekaniec K., Kucińska A., Szczygieł J., Turek K., Gajda R., Szczepańska-Gieracha J. (2022). Effects of Immersive Virtual Therapy as a Method Supporting Recovery of Depressive Symptoms in Post-Stroke Rehabilitation: Randomized Controlled Trial. Clin. Interv. Aging.

[30] Tayyebi G., Asadiof F., Hashempour B., Lotfi M., Taheri M., Naeim M. (2025). Efficacy of Virtual Reality-Based Cognitive Behavioral Group Therapy in Enhancing Emotional Well-Being and Quality of Life in Parkinson’s Disease: A Randomized Controlled Trial. Clin. Park Relat. Disord..

[31] Appel L., Kisonas E., Appel E., Klein J., Bartlett D., Rosenberg J., Smith C.N. (2021). Administering Virtual Reality Therapy to Manage Behavioral and Psychological Symptoms in Patients with Dementia Admitted to an Acute Care Hospital: Results of a Pilot Study. JMIR Form. Res..

[32] Brimelow R.E., Thangavelu K., Beattie R., Dissanayaka N.N. (2022). Feasibility of Group-Based Multiple Virtual Reality Sessions to Reduce Behavioral and Psychological Symptoms in Persons Living in Residential Aged Care. J. Am. Med. Dir. Assoc..

[33] Appel L., Appel E., Kisonas E., Lewis S., Sheng L.Q. (2022). Virtual Reality for Veteran Relaxation: Can VR Therapy Help Veterans Living with Dementia Who Exhibit Responsive Behaviors?. Front. Virtual Real..

[34] Appel L., Appel E., Bogler O., Wiseman M., Cohen L., Ein N., Abrams H.B., Campos J.L. (2020). Older Adults with Cognitive and/or Physical Impairments Can Benefit from Immersive Virtual Reality Experiences: A Feasibility Study. Front. Med..

[35] Woo O.K.L., Lee A.M., Ng R., Eckhoff D., Lo R., Cassinelli A. (2024). Flourishing-Life-Of-Wish Virtual Reality Relaxation Therapy (FLOW-VRT-Relaxation) Outperforms Traditional Relaxation Therapy in Palliative Care: Results from a Randomized Controlled Trial. Front. Virtual Real..

[36] Wu S., Liu G., Yang J., Xie X., Wu M.-E., Wang L., Zhang Y., Chen J., Wang X., Li W. (2025). Psychological Effects of Virtual Reality Intervention on Breast Cancer Patients with Different Personalities: A Randomized Controlled Trial. Int. J. Nurs. Sci..

[37] Moon N.O., Henstridge-Blows J.R., Sprecher E.A., Thomas E., Byfield A., McGrane J. (2023). ‘Godrevy Project’: Virtual Reality for Symptom Control and Well-Being in Oncology and Palliative Care—A Non-Randomised Pre-Post Interventional Trial. BMJ Oncol..

[38] Niki K., Okamoto Y., Maeda I., Mori I., Ishii R., Matsuda Y., Takagi T., Uejima E. (2019). A Novel Palliative Care Approach Using Virtual Reality for Improving Various Symptoms of Terminal Cancer Patients: A Preliminary Prospective, Multicenter Study. J. Palliat. Med..

[39] Wan Y., Gao H., Zhou K., Zhang X., Xue R., Zhang N. (2024). Virtual Reality Improves Sleep Quality and Associated Symptoms in Patients with Chronic Insomnia. Sleep Med..

[40] Malbos E., Chichery N., Borwell B., Weindel G., Molitor J., Einig-Iscain M., Seimandi J., Lançon C. (2025). Virtual Reality and Relaxation for the Treatment of Generalized Anxiety Disorder: A Randomized Comparative Study with Standard Intervention. J. Clin. Med..

[41] Cieślik B., Juszko K., Kiper P., Szczepańska-Gieracha J. (2023). Immersive Virtual Reality as Support for the Mental Health of Elderly Women: A Randomized Controlled Trial. Virtual Real..

[42] Lee S.Y., Kang J. (2020). Effect of Virtual Reality Meditation on Sleep Quality of Intensive Care Unit Patients: A Randomised Controlled Trial. Intensive Crit Care Nurs..

[43] Chan J.Y.C., Chan T.K., Wong M.P.F., Cheung R.S.M., Yiu K.K.L., Tsoi K.K.F. (2020). Effects of Virtual Reality on Moods in Community Older Adults. A Multicenter Randomized Controlled Trial. Int. J. Geriatr. Psychiatry.

[44] Şansal K.E., Şimşek A.C., Aktan S., Özbey F., Paksoy A. (2024). Restorative Effects of Virtual Nature on the Emotional Well-Being of Community-Dwelling Older Adults. Eur. J. Geriatr. Gerontol..

[45] Fan C.-C., Choy C.-S., Huang C.-M., Chih P.-S., Lee C.-C., Lin F.-H., Guo J.-L. (2022). The Effects of a Combination of 3D Virtual Reality and Hands-on Horticultural Activities on Mastery, Achievement Motives, Self-Esteem, Isolation and Depression: A Quasi-Experimental Study. BMC Geriatr..

[46] Sadowski I., Meilleur-Bédard M., Khoury B. (2025). A Novel Virtual Reality-Based Nature Meditation Program for Older Adults’ Mental Health: Results from a Pilot Randomized Controlled Trial. Clin. Gerontol..

[47] Khirallah Abd El Fatah N., Abdelwahab Khedr M., Alshammari M., Mabrouk Abdelaziz Elgarhy S. (2024). Effect of Immersive Virtual Reality Reminiscence versus Traditional Reminiscence Therapy on Cognitive Function and Psychological Well-Being among Older Adults in Assisted Living Facilities: A Randomized Controlled Trial. Geriatr. Nurs..

[48] Chaze F., Hayden L., Azevedo A., Kamath A., Bucko D., Kashlan Y., Dube M., De Paula J., Jackson A., Reyna C. (2022). Virtual Reality and Well-Being in Older Adults: Results from a Pilot Implementation of Virtual Reality in Long-Term Care. J. Rehabil. Assist. Technol. Eng..

[49] Gruber S.-S., Weigel A., Tischendorf T., Schaal T., Hellbach S. (2022). VR in Nursing Facilities—A Randomized Controlled Multicenter Pilot Study Analyzing the Changes in the State of Mind of Seniors in Nursing Facilities through the Viewing of 360° Videos. J. Public Health.

[50] Cheng V.Y.-W., Huang C.-M., Liao J.-Y., Hsu H.-P., Wang S.-W., Huang S.-F., Guo J.-L. (2020). Combination of 3-Dimensional Virtual Reality and Hands-On Aromatherapy in Improving Institutionalized Older Adults’ Psychological Health: Quasi-Experimental Study. J. Med. Internet Res..

[51] Xiaoxue S., Huang X. (2024). Promoting Stress and Anxiety Recovery in Older Adults: Assessing the Therapeutic Influence of Biophilic Green Walls and Outdoor View. Front. Public Health.

[52] Burrows B.T., Morgan A.M., King A.C., Hernandez R., Wilund K.R. (2023). Virtual Reality Mindfulness and Personalized Exercise for Patients on Hemodialysis with Depressive Symptoms: A Feasibility Study. Kidney Dial..

[53] Stevenson M.P., Schilhab T., Bentsen P. (2018). Attention Restoration Theory II: A Systematic Review to Clarify Attention Processes Affected by Exposure to Natural Environments. J. Toxicol. Environ. Health Part B Crit. Rev..

[54] Ohly H., White M.P., Wheeler B.W., Bethel A., Ukoumunne O.C., Nikolaou V., Garside R. (2016). Attention Restoration Theory: A Systematic Review of the Attention Restoration Potential of Exposure to Natural Environments. J. Toxicol. Environ. Health Part B.

[55] Flannery M. (2017). Self-Determination Theory: Intrinsic Motivation and Behavioral Change. Oncol. Nurs. Forum.

[56] Scheepers D., Keller J. (2022). On the Physiology of Flow: Bridging Flow Theory with the Biopsychosocial Model of Challenge and Threat. Int. J. Psychophysiol..

[57] Nilsson N.C., Nordahl R., Serafin S. (2016). Immersion Revisited: A Review of Existing Definitions of Immersion and Their Relation to Different Theories of Presence. Hum. Technol..

[58] Slater M. (2003). A Note on Presence Terminology. Presence Connect..

[59] Cummings J.J., Bailenson J.N. (2016). How Immersive Is Enough? A Meta-Analysis of the Effect of Immersive Technology on User Presence. Media Psychol..

[60] Kubr J., Lochmannová A., Hořejší P. (2024). Immersive Virtual Reality Training in Industrial Settings: Effects on Memory Retention and Learning Outcomes. IEEE Access.

[61] Goto S., Park B.-J., Tsunetsugu Y., Herrup K., Miyazaki Y. (2013). The Effect of Garden Designs on Mood and Heart Output in Older Adults Residing in an Assisted Living Facility. HERD.

[62] Taylor E.M., Robertson N., Lightfoot C.J., Smith A.C., Jones C.R. (2022). Nature-Based Interventions for Psychological Wellbeing in Long-Term Conditions: A Systematic Review. Int. J. Environ. Res. Public Health.

[63] Jo H., Song C., Miyazaki Y. (2019). Physiological Benefits of Viewing Nature: A Systematic Review of Indoor Experiments. Int. J. Environ. Res. Public Health.

[64] Wen Y., Shen X., Shen Y. (2024). Improving Immersive Experiences in Virtual Natural Setting for Public Health and Environmental Design: A Systematic Review and Meta-Analysis of Randomized Controlled Trials. PLoS ONE.

[65] Zhang Y., Zhang L., Hua H., Jin J., Zhu L., Shu L., Xu X., Kuang F., Liu Y. (2021). Relaxation Degree Analysis Using Frontal Electroencephalogram Under Virtual Reality Relaxation Scenes. Front. Neurosci..

[66] de Zambotti M., Yuksel D., Kiss O., Barresi G., Arra N., Volpe L., King C., Baker F.C. (2022). A Virtual Reality-Based Mind–Body Approach to Downregulate Psychophysiological Arousal in Adolescent Insomnia. Digit Health.

[67] Yang H., Herbelin B., Ngo C., Vuarnesson L., Blanke O. (2025). Meditation in the Third-Person Perspective Modulates Minimal Self and Heartbeat-Evoked Potentials. NeuroImage.

[68] Jo M., Kim E., Lee J. (2024). Virtual Reality vs. Imagery: Comparing Approaches in Guided Meditation. Front. Psychol..

[69] Askay S.W., Patterson D.R., Sharar S.R. (2009). VIRTUAL REALITY HYPNOSIS. Contemp. Hypn..

[70] Zhao F.-Y., Li L., Xu P., Kennedy G.A., Zheng Z., Wang Y.-M., Zhang W.-J., Yue L.-P., Ho Y.-S., Fu Q.-Q. (2024). Mapping Knowledge Landscapes and Evolving Trends of Clinical Hypnotherapy Practice: A Bibliometrics-Based Visualization Analysis. IJGM.

[71] Walter N., Leyva M.T., Hinterberger T., Rupp M., Loew T., Lambert-Delgado A., Mena A.E.C. (2025). Hypnosis as a Non-Pharmacological Intervention for Invasive Medical Procedures—A Systematic Review and Meta-Analytic Update. J. Psychosom. Res..

[72] Yagihashi M., Sakuma A., Murakami M. (2025). Psychotherapies and Psychological Support for Individuals Facing Psychological Distress during the COVID-19 Pandemic: A Scoping Review. PLoS ONE.

[73] Blackmore R., Giles C., Tremain H., Kelly R., Foley F., Fletcher K., Nedeljkovic M., Wadley G., Seabrook E., Thomas N. (2024). Examining the Use of Virtual Reality to Support Mindfulness Skills Practice in Mood and Anxiety Disorders: Mixed Methods Study. J. Med. Internet Res..

[74] Shankar R., Bundele A., Mukhopadhyay A. (2025). The Effectiveness of Virtual Reality-Based Mindfulness Interventions for Managing Stress, Anxiety, and Depression: Protocol for a Systematic Review and Meta-Analysis of Randomized Controlled Trials. JMIR Res. Protoc..

[75] Pardini S., Calcagno R., Genovese A., Salvadori E., Ibarra O.M. (2025). Exploring Virtual Reality-Based Reminiscence Therapy on Cognitive and Emotional Well-Being in People with Cognitive Impairments: A Scoping Review. Brain Sci..

[76] Ng W.H.D., Ang W.H.D., Fukahori H., Goh Y.S., Lim W.S., Siah C.J.R., Seah B., Liaw S.Y. (2024). Virtual Reality-Based Reminiscence Therapy for Older Adults to Improve Psychological Well-Being and Cognition: A Systematic Review. J. Clin. Nurs..

[77] Ma H., Wu Q., Hu K., Liu J., Huang Y., Liu X., Yang Q. (2025). Scoping Review of Gamification in Rehabilitation Care of Adults with Chronic Illnesses. Nurs. Res..

[78] Kim H., Choi Y. (2025). Developing Interactive VR-Based Digital Therapeutics for Acceptance and Commitment Therapy (ACT): A Structured Framework for the Digital Transformation Integrating Gamification and Multimodal Arts. Front. Psychiatry.

[79] Slater M. (2018). Immersion and the Illusion of Presence in Virtual Reality. Br. J. Psychol..

[80] Mazgelytė E., Zagorskaja J., Dereškevičiūtė E., Petrėnas T., Kaminskas A., Songailienė J., Utkus A., Chomentauskas G., Karčiauskaitė D. (2022). Dynamics of Physiological, Biochemical and Psychological Markers during Single Session of Virtual Reality-Based Respiratory Biofeedback Relaxation. Behav. Sci..

[81] Kim H.-G., Cheon E.-J., Bai D.-S., Lee Y.H., Koo B.-H. (2018). Stress and Heart Rate Variability: A Meta-Analysis and Review of the Literature. Psychiatry Investig..

[82] Lomas T., Ivtzan I., Fu C.H. (2015). A Systematic Review of the Neurophysiology of Mindfulness on EEG Oscillations. Neurosci. Biobehav. Rev..

[83] Drazich B.F., McPherson R., Gorman E.F., Chan T., Teleb J., Galik E., Resnick B. (2023). In Too Deep? A Systematic Literature Review of Fully-Immersive Virtual Reality and Cybersickness among Older Adults. J. Am. Geriatr. Soc..

[84] Kalantari S., Bill Xu T., Mostafavi A., Lee A., Barankevich R., Boot W.R., Czaja S.J. (2022). Using a Nature-Based Virtual Reality Environment for Improving Mood States and Cognitive Engagement in Older Adults: A Mixed-Method Feasibility Study. Innov. Aging.

[85] Oh S.S., Kim K.-A., Kim M., Oh J., Chu S.H., Choi J. (2021). Measurement of Digital Literacy Among Older Adults: Systematic Review. J. Med. Internet Res..

[86] Felber N.A., Mihailov E., Wangmo T. (2025). Virtual Reality as a Possible Aged Care Technology—Opportunities and Prejudices from Older Persons and Their Caregivers in a Qualitative Study. Front. Virtual Real..

[87] Madary M., Metzinger T.K. (2016). Real Virtuality: A Code of Ethical Conduct. Recommendations for Good Scientific Practice and the Consumers of VR-Technology. Front. Robot. AI.

[88] Kalantari S., Xu T.B., Mostafavi A., Kim B., Dilanchian A., Lee A., Boot W.R., Czaja S.J. (2023). Using Immersive Virtual Reality to Enhance Social Interaction Among Older Adults: A Cross-Site Investigation. Innov. Aging.

[89] Tichko P., Kim J.C., Large E., Loui P. (2022). Integrating Music-Based Interventions with Gamma-Frequency Stimulation: Implications for Healthy Ageing. Eur. J. Neurosci..

[90] Chaddock-Heyman L., Loui P., Weng T.B., Weisshappel R., McAuley E., Kramer A.F. (2021). Musical Training and Brain Volume in Older Adults. Brain Sci..

[91] Neuhaus C., Schneider A. (2017). Methods in Neuromusicology: Principles, Trends, Examples and the Pros and Cons. Studies in Musical Acoustics and Psychoacoustics.

[92] Blum J., Rockstroh C., Göritz A.S. (2019). Heart Rate Variability Biofeedback Based on Slow-Paced Breathing with Immersive Virtual Reality Nature Scenery. Front. Psychol..

[93] Mazgelytė E., Rekienė V., Dereškevičiūtė E., Petrėnas T., Songailienė J., Utkus A., Chomentauskas G., Karčiauskaitė D. (2021). Effects of Virtual Reality-Based Relaxation Techniques on Psychological, Physiological, and Biochemical Stress Indicators. Healthcare.

