# Supplementary material for: Mechanisms of Virtual Reality-Based Relaxation in Older Adults: A Scoping Review
Source: J Clin Med. 2025 Aug 29;14(17):6126. doi: 10.3390/jcm14176126 (PMC12429708; doi:10.3390/jcm14176126)
Supplement: Supplementary file 1 [file jcm-14-06126-s001.zip › Supplementary File S3..pdf]

Supplementary File S3. List of excluded studies with reason

Incorrect participants:

1. Tan HLE, Chng CML, Lau Y, Klainin-Yobas P. Investigating the effects of a virtual reality-based stress management programme on inpatients with mental disorders: A pilot randomised controlled trial. Int J Psychol. 2021;56(3):444-453. doi:10.1002/ijop.12713
2. Maier M, Ballester BR, Leiva Bañuelos N, Duarte Oller E, Verschure PFMJ. Adaptive conjunctive cognitive training (ACCT) in virtual reality for chronic stroke patients: a randomized controlled pilot trial. J Neuroeng Rehabil. 2020;17(1):42. Published 2020 Mar 6. doi:10.1186/s12984-020-0652-3
3. Bachelard L, Blanc N, Michel A. Immersive virtual nature for improving psychological distress of breast cancer patients undergoing intravenous chemotherapy. Support Care Cancer. 2025;33(7):541. Published 2025 Jun 5. doi:10.1007/s00520-025-09563-x
4. Gerber SM, Jeitziner MM, Wyss P, et al. Visuo-acoustic stimulation that helps you to relax: A virtual reality setup for patients in the intensive care unit. Sci Rep. 2017;7(1):13228. Published 2017 Oct 16. doi:10.1038/s41598-017-13153-1
5. Ucero-Lozano R, Donoso-Úbeda E, Cuesta-Barriuso R, Pérez-Llanes R. Immersive VR movement visualization in patients with hemophilic knee arthropathy: randomized, multicenter, single-blind clinical trial. Disabil Rehabil. 2025;47(11):2770-2777. doi:10.1080/09638288.2024.2401138
6. Lotfinia S, Yaseri A, Jamshidmofid P, et al. Effect of Relaxation-Based Virtual Reality on Psychological and Physiological Stress of Substance Abusers Under Detoxification: A Randomized Controlled Trial. Brain Behav. 2024;14(10):e70084. doi:10.1002/brb3.70084
7. Merliot-Gailhoustet L, Raimbert C, Garnier O, et al. Discomfort improvement for critically ill patients using electronic relaxation devices: results of the cross-over randomized controlled trial E-CHOISIR (Electronic-CHOIce of a System for Intensive care Relaxation) [published correction appears in Crit Care. 2022 Sep 26;26(1):291. doi: 10.1186/s13054-022-04169-9.]. *Crit Care*. 2022;26(1):263. Published 2022 Sep 3. doi:10.1186/s13054-022-04136-4
8. Marshall J, Devane N, Talbot R, et al. A randomised trial of social support group intervention for people with aphasia: A Novel application of virtual reality. *PLoS One*. 2020;15(9):e0239715. Published 2020 Sep 24. doi:10.1371/journal.pone.0239715
9. Yang JH, Ryu JJ, Nam E, Lee HS, Lee JK. Effects of Preoperative Virtual Reality Magnetic Resonance Imaging on Preoperative Anxiety in Patients Undergoing Arthroscopic Knee Surgery: A Randomized Controlled Study. Arthroscopy. 2019;35(8):2394-2399. doi:10.1016/j.arthro.2019.02.037
10. Stansel CC, McLeod AR, Gulati S, et al. Effects of Virtual Reality on Pain, Stress, and Affect in an Outpatient Chemotherapy Infusion Clinic: A Randomized Controlled Trial. Clin J Oncol Nurs. 2025;29(1):65-71. doi:10.1188/25.CJON.65-71

Incorrect intervention:

11. Shannon MM, White M, Churilov L, et al. Re-Imagining Hospital Patient Room Design for People After stroke: A Randomized Controlled Study Using Virtual Reality. Stroke. 2024;55(7):1895-1903. doi:10.1161/STROKEAHA.124.046252

12. Barsasella D, Liu MF, Malwade S, et al. Effects of Virtual Reality Sessions on the Quality of Life, Happiness, and Functional Fitness among the Older People: A Randomized Controlled Trial from Taiwan. Comput Methods Programs Biomed. 2021;200:105892. doi:10.1016/j.cmpb.2020.105892
13. Fidan O, Kiloatar H, Colak E, Oskay D. Effects of group-based virtual reality training on activities of daily living and functional outcomes in older adults: a randomised control trial. Disabil Rehabil Assist Technol. 2025;20(4):1044-1055. doi:10.1080/17483107.2024.2431051
14. Tang TW, Wang TC, Tsai CL. The influence of different tree densities on alpha waves, physical activity enjoyment, and satisfaction of late middle-aged and older adults using virtual cycling. Exp Gerontol. 2024;197:112608. doi:10.1016/j.exger.2024.112608
15. Gautama MSN, Haryani H, Huang TW, Chen JH, Chuang YH. Effectiveness of smartphone-based virtual reality relaxation (SVR) for enhancing comfort in cancer patients undergoing chemotherapy: a randomized controlled trial. Support Care Cancer. 2024;32(12):824. Published 2024 Nov 26. doi:10.1007/s00520-024-09036-7
16. Burin D, Kawashima R. Repeated Exposure to Illusory Sense of Body Ownership and Agency Over a Moving Virtual Body Improves Executive Functioning and Increases Prefrontal Cortex Activity in the Elderly. Front Hum Neurosci. 2021;15:674326. Published 2021 May 31. doi:10.3389/fnhum.2021.674326
17. Lin RC, Chiang SL, Heitkemper MM, et al. Effectiveness of Early Rehabilitation Combined With Virtual Reality Training on Muscle Strength, Mood State, and Functional Status in Patients With Acute Stroke: A Randomized Controlled Trial. Worldviews Evid Based Nurs. 2020;17(2):158-167. doi:10.1111/wvn.12429
18. Cameirão MS, Faria AL, Paulino T, Alves J, Bermúdez I Badia S. The impact of positive, negative and neutral stimuli in a virtual reality cognitive-motor rehabilitation task: a pilot study with stroke patients. J Neuroeng Rehabil. 2016;13(1):70. Published 2016 Aug 9. doi:10.1186/s12984-016-0175-0
19. Kim DR, Song S, Kim GM, et al. Effects of ICT-Based Multicomponent Program on Body Composition and Cognitive Function in Older Adults: A Randomized Controlled Clinical Study. Clin Interv Aging. 2021;16:1161-1171. Published 2021 Jun 21. doi:10.2147/CIA.S306894
20. Tominari, M., Uozumi, R., Becker, C., & Kinoshita, A. (2021). Reminiscence therapy using virtual reality technology affects cognitive function and subjective well-being in older adults with dementia. Cogent Psychology, 8(1). https://doi.org/10.1080/23311908.2021.1968991
21. Rousseaux F, Dardenne N, Massion PB, et al. Virtual reality and hypnosis for anxiety and pain management in intensive care units: A prospective randomised trial among cardiac surgery patients. *Eur J Anaesthesiol*. 2022;39(1):58-66. doi:10.1097/EJA.0000000000001633
22. Vlake JH, van Bommel J, Wils EJ, et al. Intensive Care Unit-Specific Virtual Reality for Critically Ill Patients With COVID-19: Multicenter Randomized Controlled Trial. *J Med Internet Res*. 2022;24(1):e32368. Published 2022 Jan 31. doi:10.2196/32368
23. Stamm O, Dahms R, Müller-Werdan U. Virtual reality in pain therapy: a requirements analysis for older adults with chronic back pain. *J Neuroeng Rehabil*. 2020;17(1):129. Published 2020 Sep 29. doi:10.1186/s12984-020-00753-8
24. Vieira Á, Melo C, Machado J, Gabriel J. Virtual reality exercise on a home-based phase III cardiac rehabilitation program, effect on executive function, quality of life and

depression, anxiety and stress: a randomized controlled trial. Disabil Rehabil Assist Technol. 2018;13(2):112-123. doi:10.1080/17483107.2017.1297858

Incorrect outcomes:

25. Cao W, Ren F, Li T, et al. Effect of virtual reality intervention on hospitalized patients with acute pain after thoracoscopic surgery: a pilot randomized clinical trial. Int J Surg. 2025;111(3):2752-2756. Published 2025 Mar 1. doi:10.1097/JS9.0000000000002264
26. Sariköse A, Turan GB. The Effects of Virtual Reality Application on Pain Intensity, Anxiety Level and Patient Satisfaction in Patients Who Undergo Bronchoscopy: A Randomized Controlled Trial. Pain Manag Nurs. 2025;26(1):102-110. doi:10.1016/j.pmn.2024.10.007
27. Makmee P, Wongupparaj P. Virtual Reality-based Cognitive Intervention for Enhancing Executive Functions in Community-dwelling Older Adults. Psychosoc Interv. 2022;31(3):133-144. Published 2022 Jul 20. doi:10.5093/pi2022a10
28. Le Du K, Septans AL, Maloisel F, et al. A New Option for Pain Prevention Using a Therapeutic Virtual Reality Solution for Bone Marrow Biopsy (REVEH Trial): Open-Label, Randomized, Multicenter, Phase 3 Study. *J Med Internet Res*. 2023;25:e38619. Published 2023 Feb 15. doi:10.2196/38619
29. Perra A, Galetti A, Zaccheddu R, Locci A, Piludu F, Preti A, Primavera D, Di Natale L, Nardi AE, Kurotshka PK, et al. A Recovery-Oriented Program for People with Bipolar Disorder through Virtual Reality-Based Cognitive Remediation: Results of a Feasibility Randomized Clinical Trial. *Journal of Clinical Medicine*. 2023; 12(6):2142. https://doi.org/10.3390/jcm12062142

Incorrect design:

30. Szczepocka E, Mokros Ł, Kazmierski J, et al. The Effectiveness of Virtual Reality-Based Training on Cognitive, Social, and Physical Functioning in High-Functioning Older Adults (CoSoPhy FX): 2-Arm, Parallel-Group Randomized Controlled Trial. *JMIR Res Protoc*. 2024;13:e53261. Published 2024 Jun 5. doi:10.2196/53261
31. Kwan RYC, Ng F, Lam LCW, Yung RC, Sin OSK, Chan S. The effects of therapeutic virtual reality experience to promote mental well-being in older people living with physical disabilities in long-term care facilities. Trials. 2023;24(1):558. Published 2023 Aug 26. doi:10.1186/s13063-023-07592-7
