# Supplementary material for: Mechanisms of Virtual Reality-Based Relaxation in Older Adults: A Scoping Review
Source: J Clin Med. 2025 Aug 29;14(17):6126. doi: 10.3390/jcm14176126 (PMC12429708; doi:10.3390/jcm14176126)
Supplement: Supplementary file 1 [file jcm-14-06126-s001.zip › Supplementary file S2.pdf]

Supplementary File 2. Search strategy for databases

PubMed: ("Aged"[MeSH Terms] OR "aged, 80 and over"[MeSH Terms] OR "elder*"[Title/Abstract] OR "senior*"[Title/Abstract] OR "older adult*"[Title/Abstract] OR "geriatric*"[Title/Abstract]) AND ("Virtual Reality"[MeSH Terms] OR "Virtual Reality Exposure Therapy"[MeSH Terms] OR "immersive"[Title/Abstract] OR "HMD"[Title/Abstract] OR "vr headset*"[Title/Abstract] OR "Virtual Reality"[Title/Abstract] OR "total immersion"[Title/Abstract]) AND ("Relaxation"[MeSH Terms] OR "Relaxation Therapy"[MeSH Terms] OR "stress, psychological"[MeSH Terms] OR "Mindfulness"[MeSH Terms] OR "relax*"[Title/Abstract] OR "stress reduction"[Title/Abstract] OR "mood"[Title/Abstract] OR "well-being"[Title/Abstract]) AND ("Randomized Controlled Trial"[Publication Type] OR "Clinical Trial"[Publication Type] OR "randomized"[Title/Abstract] OR "randomised"[Title/Abstract] OR "quasi random*"[Title/Abstract] OR "non random*"[Title/Abstract])

SCOPUS: (TITLE-ABS-KEY (aged OR "aged 80 and over" OR elder* OR senior* OR "older adult*" OR geriatric*)) AND (TITLE-ABS-KEY ("virtual reality" OR "head-mounted display" OR HMD OR immersive OR "VR headset*" OR "total immersion")) AND (TITLE-ABS-KEY (relaxation OR "relaxation therapy" OR mindfulness OR "stress reduction" OR mood OR "well-being")) AND (TITLE-ABS-KEY (randomized OR randomised OR RCT OR "clinical trial" OR "quasi-random*" OR "non-random*"))

WOS: (((ALL=("Adult" OR "elder*" OR "senior" OR "older" OR "middle aged")) AND ALL=("Virtual Reality" OR "Virtual Reality Exposure Therapy" OR "head-mounted display" OR "HMD" OR "VRET" OR "total immersion" OR "virtual reality" OR "virtual reality exposure therapy" OR "VR headset" OR "VR")) AND ALL=("Relaxation" OR "Relaxation Therapy" OR "Muscle Relaxation" OR "Depression" OR "Depressive Disorder" OR "Mood Disorders" OR "Stress, Psychological" OR "Mindfulness" OR "Psychotherapy" OR "Mental Health" OR "stress" OR "depression" OR "depressive symptoms" OR "mental health")) AND ALL=("Randomized clinical trial" OR "RCT" OR "random*" OR "Randomized controlled trial" OR "Randomised clinical trial" OR "randomised" OR "Randomised controlled trial" OR "quasi-random*" OR "non random*" OR "non-random*" OR "clinical study" OR "clinical trial")

Cochrane

	**Terms**	**Hits**
#1	MeSH descriptor: [Aged] explode all trees	281613
#2	MeSH descriptor: [Aged, 80 and over] explode all trees	71266
#3	(elder* OR senior* OR geriatric*)	87646
#4	(older NEXT adult*)	23352

#5	#1 OR #2 OR #3 OR #4	350907
#6	MeSH descriptor: [Virtual Reality] explode all trees	1478
#7	immersive OR HMD	2067
#8	VR NEXT headset*	325
#9	head-mounted NEXT display	464
#10	total NEXT immersion	9
#11	virtual NEXT reality	8690
#12	#6 OR #7 OR #8 OR #9 OR #10 OR #11	9236
#13	MeSH descriptor: [Relaxation Therapy] explode all trees	2740
#14	MeSH descriptor: [Mindfulness] explode all trees	2531
#15	(relax*)	25418
#16	(stress NEXT reduction)	3206
#17	(mindfulness)	11379
#18	(mood)	30419
#19	(well NEXT being)	25617
#20	#13 OR #14 OR #15 OR #16 OR #17 OR #18 OR #19	85306
Final	#5 AND #12 AND #20	171
